# The complete mitochondrial genome and phylogenetic analysis of *Pealius mori* (Hemiptera: Aleyrodidae)

**DOI:** 10.1080/23802359.2024.2373229

**Published:** 2024-07-05

**Authors:** Yonghong Zhang, Jianping Chai, Zhenguo Yang, Jiafu Luo

**Affiliations:** Sericulture and Apiculture Research Institute, Yunnan Academy of Agricultural Science, Mengzi, Yunnan, China

**Keywords:** *Pealius mori*, aleyrodidae, mitochondrion, phylogenetic tree

## Abstract

The complete mitochondrial genome of *Pealius mori* (Hemiptera: Aleyrodidae) was determined in this study. The mitogenome was 15,654 bp long with 37 typical Insecta mitochondrial genes and one non-coding control region. Its gene content and order were different to other Hemiptera mitochondrial genomes. The overall nucleotide composition of the mitogenome was 42.62% A, 32.73% T, 11.12% G and 13.54% C, with an A + T bias of 75.34%. Phylogenetic analyses of 14 species in Aleyrodidae, 2 species in Lepidoptera and 1 species in Thysanoptera by Maximum Likelihood showed that *P. mori* China had been more closely related to *P. mori* France, closely related to *Pealius machili*. This result well supported the taxonomic position of Aleyrodidae and their close relationship with the *Pealius* category.

## Introduction

*Pealius mori* (Takahashi 1932) belongs to the Hemiptera Aleyrodidae whitefly family and is important whitefly pests that pose a significant threat to agricultural production in China (Xiong et al. 2011). The mulberry whitefly (*P.mori* Takahashi) is one of the six main species of whiteflies that cause damage to crops in China (Tao et al. 2012). *P. mori* are widely distributed around the sericulture areas (David and Ragupathy 2004; Maketon et al. 2009; Wang et al. 2016). The mulberry whitefly harms mulberry leaves by sucking on their juice through larvae and adults, and its secretions lead to the prevalence of coal pollution in mulberry gardens, resulting in a decrease in the quality and yield of mulberry leaves. *P. mori* is distributed in various mulberry planting areas in China, with a wide range of host plants (Suh et al. 2008; Jiang et al. 2014). The factors that affect the control effect of the mulberry whitefly are very complex due to its small size, overlapping generations, and strong migration ability of adults (Gao et al. 1998). Studying the genetic structure of pest populations, revealing their dispersal routes and occurrence trends, is beneficial for adopting comprehensive technical measures for effective prevention and control.

In 1939, *P. mori* was reported initially in Chinese sericultural areas, it had also caused harm in Yunnan silkworm-raising regions and has been increasing year by year. *P. mori* Takahashi populations were widely distributed in different sericultural areas of Yunnan Province, they were analyzed using mitochondrial cytochrome c oxidase I (mtCOI) and microsatellite marker (Chai et al. 2022; Jiang et al. 2023), there were seven geographical populations of *P. mori* Takahashi present in Yunnan sericulture areas. Here, we reported the complete mitochondrial genome of *P. mori* and investigated its phylogenetic position with the related taxa in the family Aleyrodidae.

## Materials and methods

**Figure 1.**
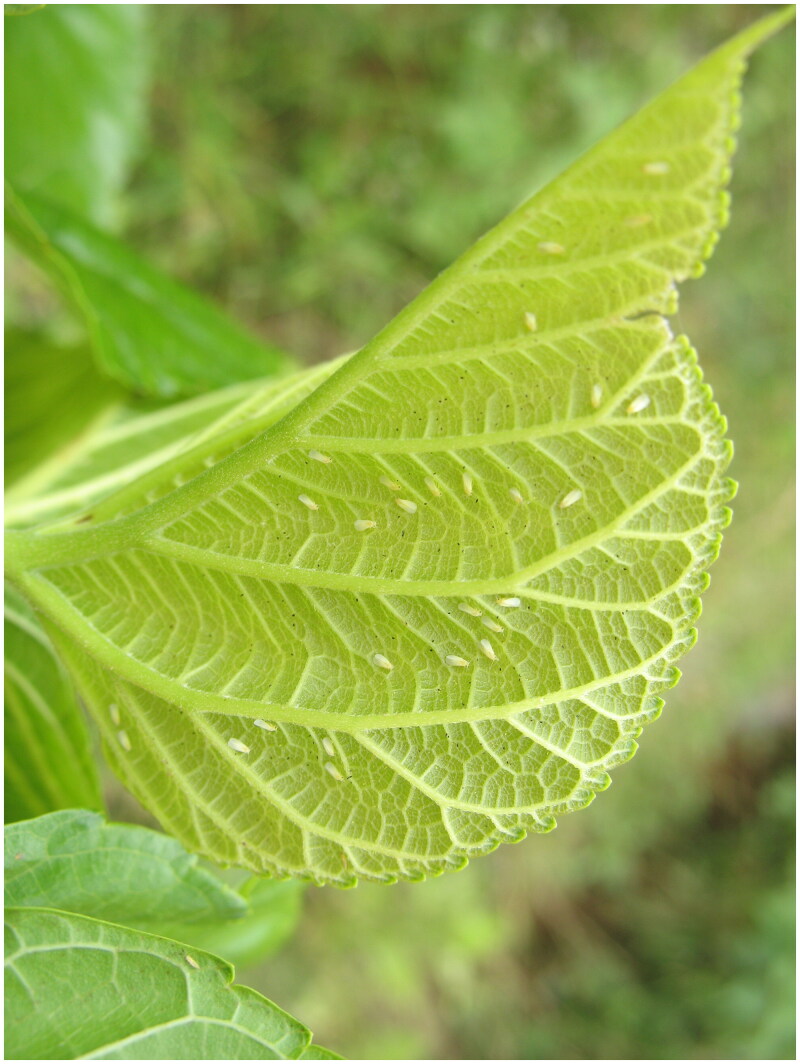
A Photo of the *Pealius mori*. Photograph was taken by Jianping Chai.

The adult of *P. mori* was collected from mulberry leaves in the Caoba Town, Mengzi City, Honghe Autonomous Prefecture, Yunnan Province, China (23°31′13″N, 103°23′52″E) (Figure 1). The sample was amassed by Jiafu Luo. The specimen (voucher number: YNSFS01) was used for extracting the genomic DNA and its DNA were stored in molecular biology laboratory of Sericulture and Apiculture Research Institute, Yunnan Academy of Agricultural Science (https://www.yaas.org.cn, Xingrong Bai, bxrong3@163.com). The genomic DNA used for protein K-SDS-phenol/chloroform extraction method (Zhang et al. 2019). The extractive genome was sent to Sangon Biotech (Shanghai, China) for sequencing, DNA library was constructed and performed paired-end using the Illumina Miseq platform (Illumina Inc., San Diego, CA). The sequencing results were assembled using A5-miseq V20150522 and SPAdes 3.9.0 software for second-generation sequencing data (Bankevich et al. 2012; Coil et al. 2015); The read coverage depth map was shown in Figure S1. Then use Gap Filler to supplement GAP on the concatenated contig; Finally, PrInSeS-G was used for sequence correction to correct editing errors and missing insertion of small segments during the splicing process. The homology of nucleotide sequence was aligned using DNAMAN 6.0; Sequence alignment of *CO* I gene was performed by GeneDoc software. The phylogenetic relationship was recovered by the Maximum Likelihood method in IQ-TREE v.2.1.2 (Nguyen et al. 2015), based on complete mitochondrial genomes, *Pseudodendrothrips mori* was used as outgroup. Bootstrap analysis was performed with 1000 replications.

## Results

**Figure 2.**
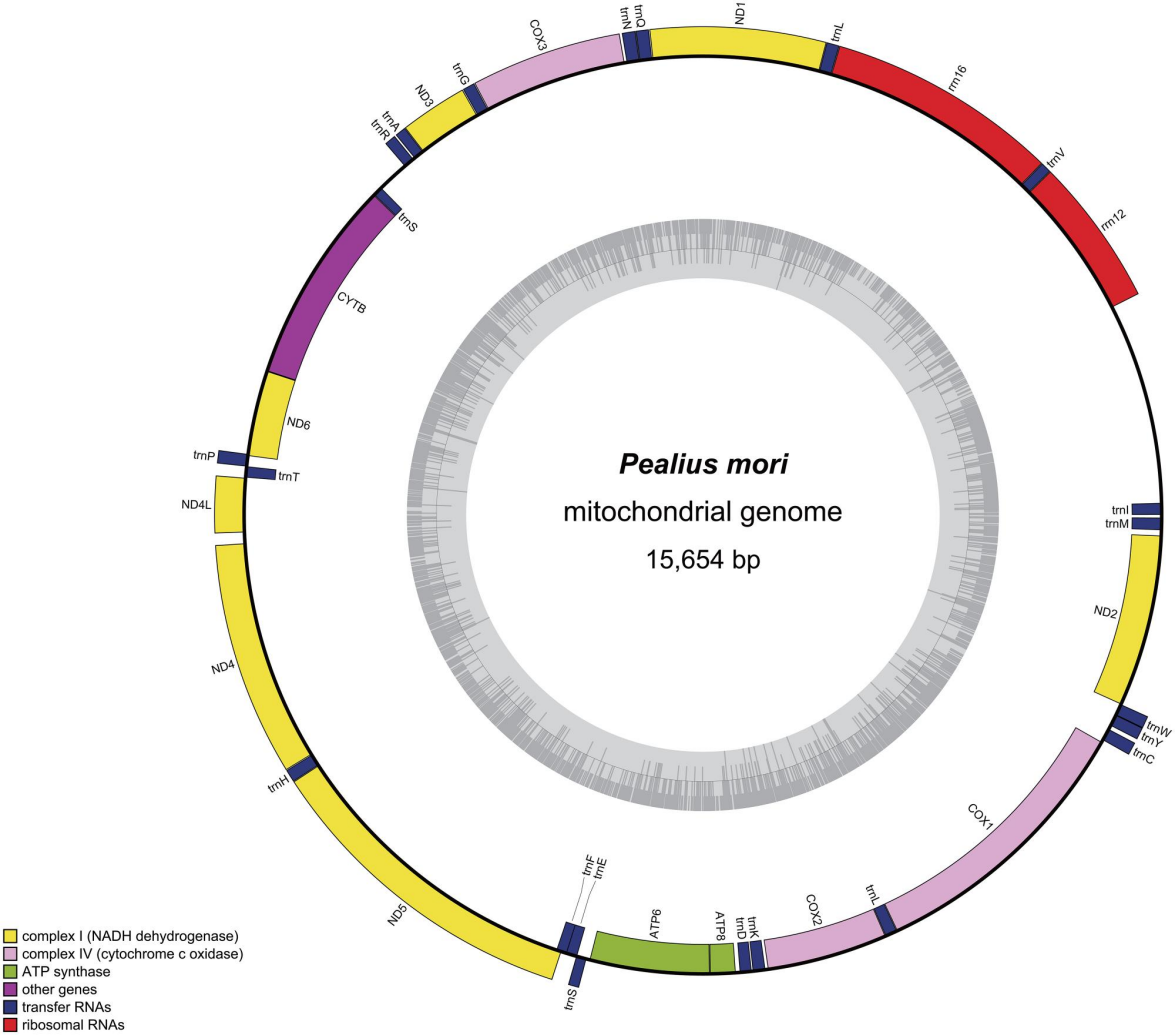
The circular mitogenome map of *Pealius mori.*

The whole mitochondrion of *P. mori* has a closed circular with 15,654 bp in length (GenBank accession no. OR759422), and encoded 37 genes, including 13 protein-coding genes (PCGs) (10,844 bp in total), 22 tRNA genes, 2 rRNA genes, and one non-coding control region (Figure 2), 37 genes correspond to the distribution of mtDNA gene positions in Hemiptera insects (Thao et al. 2004). The A + T and G + C content of the mitochondrial genome were 75.34% and 24.66%, respectively. There were 21 gene spacer regions in the mitogenome with the longest region (1103 bp) between trnI (GAU) and rrnS. 12 gene overlapping regions were observed which the regions dispersed in neighboring genes with the length varying from 1 to 24 bp, the longest overlapping nucleotide fragment was occurred between trnL (CUN) and rrnL (24 bp). Excepting *CO* I started with TTG, the initiator codons of the other 12 PCGs were ATN. The terminator codons of 11 PCGs were TAA, ND5 used TAG as the stop condon, *CO* I ended with a single T. Twenty-tw-o tRNA genes range from 55 to 71 bp in length and display average A + T content of 81.64%. The two rRNA genes, rrnL and rrnS both mapped on the N-strand, were 709 bp and 774 bp in length.

**Figure 3.**
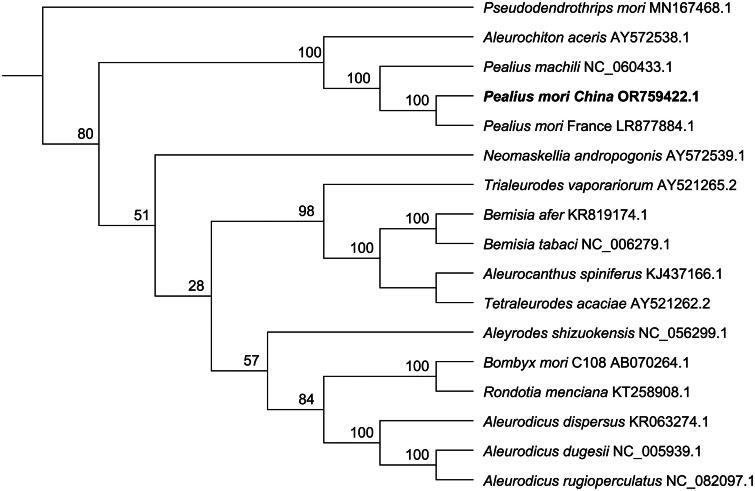
Maximum-likelihood phylogenetic tree based on mitochondrial genome sequences. *Pseudodendrothrips mori* was used as outgroup. The bold font indicated *P. mori* China came from this study. Numbers associated with the branches are bootstrap values (1000 replicates). References used for comparative analysis were provided in Table S2.

Phylogenetic analysis revealed that *P. mori* China is more closely related to *P. mori* France (Figure 3). The ecotype of *P. mori* France was *Ficus benjamina* population, and ecotype of *P. mori* China was *Morus alba* population, they were closely related to *Pealius machili, P. mori* China, *P. mori* France and *P. machili* belonged to insect of *Pealius* genus.

## Discussion and conclusion

There are differences in the mitochondrial genomes between the *P. mori* China and *P. mori* France. Firstly, there was a difference in the length of genome, 15, 654 bp and 15, 101 bp for the *P. mori* China and *P. mori* France, respectively. Furthermore, the *P. mori* China has 22 tRNA genes in its genome, while the *P. mori* France only has 20 tRNA genes. There were significant differences in non-coding regions between the two isolates. The nucleotide sequences of 12 PCGs from two strains are highly homologous (Tabel S1), while there was a significant difference in the *CO* I gene (Figure S2). The genetic diversity of species was closely related to their evolutionary potential and environmental adapt ability (Wenzel et al. 2015). *P. mori* China and *P. mori* France were different geographical populations, and their relatively independent geographical environments resulted in genetic differentiation of the mulberry whitefly population. *CO* I gene is an ideal DNA barcode for insect classification and identification. The difference in the source of host plants between the two isolates, which resulted in the two isolates being geographical subspecies. Therefore, there were significant differences in the *CO* I gene sequence; The *CO* I gene had a certain evolutionary rate within the insect mitochondrial genome, and some regions of the gene sequence were highly conserved, while some regions showed significant differences among different species (Lunt et al. 1996; Boykin et al. 2007). For the whitefly *Bemisia tabaci*, the range of genetic difference of the mtCOI sequence was 0%–34% from different population complex (Dinsdale et al. 2010).

*Pealius mori* was recorded for the first time in Greece (Wang et al. 2016), *P. mori* Takahashi populations were widely distributed in different sericultural areas of China (Abd-Rabou and Evans 2013). In Yunnan Province of China, *P. mori* Takahashi populations were analyzed using mitochondrial cytochrome c oxidase I (mtCOI), at least seven clades of *P. mori* were found in Yunnan Province (Chai et al. 2022). At present, the research of *P. mori* mainly focuses on three aspects: morphological observation, prevention and control, and its impact on *Bombyx mori* (Yu et al. 1997; Chai et al. 2013; Abd-Rabou et al. 2019). The characteristics of *P. mori* include small polypide, overlapping generations, strong migration ability of adults, and diverse host plants (Bureekham et al. 1987). The *P. mori* has undergone different stages of evolution and development, and whether the population distributed in different silkworm areas in Yunnan Province has also undergone genetic variation. These internal factors related to the outbreak of *P. mori* in various silkworm areas are worth exploring. Chai et al. (2016) found that genetic drift had resulted in great genetic differentiation of *P. mori* populations from 7 sampling sites at the south to west of Yunnan Province, and that haplotypes of *P. mori* mtDNA *CO* I gene present obvious geographic regional population-specific distribution pattern (Chai et al. 2016). Currently, there were no reports on the complete mitochondrial genome of *P. mori* in China, it was necessary to study and report on the genome.

In this study, we reported the whole mitochondrial genome of *P. mori* and analyzed the genomic characteristics and identified its genetic status with other insect species. The mitochondrial genome of *P. mori* was 15,654 bp in length and expresses high A + T content. Phylogenetic tree revealed that *P. mori* belongs to the *Pealius* genus. The *P. mori* mitochondrial genome reported here enriches the number of Aleyrodidae whitefly mitochondrial genomes available for future research. This study will contribute to the future research on systematics classification of *Pealius* genus.

## Supplementary Material

Supplemental Material

Supplemental Material

Supplemental Material

Supplemental Material

## Data Availability

The genome sequence data that support the findings of this study are available in GenBank at https://www.ncbi.nlm.nih.gov/under accession no. OR759422. The associated BioProject, SRA, and Bio-Sample numbers were PRJNA1043077, SRR26893893, and SAMN38325672, respectively.
