# Supplementary material for: The complete mitochondrial genome and phylogenetic analysis of Pealius mori (Hemiptera: Aleyrodidae)
Source: Mitochondrial DNA B Resour. 2024 Jul 5;9(7):856–60. doi: 10.1080/23802359.2024.2373229 (PMC11229734; doi:10.1080/23802359.2024.2373229)
Supplement: Supplemental Material [file TMDN_A_2373229_SM9481.doc]

**Table S2.** List of all 17 species, GenBank accession numbers and references for sequences used to construct phylogenetic trees (Figure 3).

| Species | Accession | Reference |
| --- | --- | --- |
| *Pealius mori* China | OR759422.1 | This study |
| *Pealius mori* France | LR877884.1 | Unpublished data |
| *Pealius machili* | NC_060433.1 | Zhang et al. (2020) |
| *Aleurocanthus spiniferus* | KJ437166.1 | Unpublished data |
| *Tetraleurodes acaciae* | AY521262.2 | Thao et al. (2004a) |
| *Bemisia afer* | KR819174.1 | Wang et al. (2016) |
| *Bemisia tabaci* | NC_006279.1 | Thao et al. (2004b) |
| *Aleyrodes shizuokensis* | NC_056299.1 | Lei et al. (2021) |
| *Trialeurodes vaporariorum* | AY521265.2 | Thao et al. (2004b) |
| *Aleurochiton aceris* | AY572538.1 | Thao et al. (2004b) |
| *Neomaskellia andropogonis* | AY572539.1 | Thao et al. (2004b) |
| *Aleurodicus dispersus* | KR063274.1 | Lu et al. (2017) |
| *Aleurodicus dugesii* | NC_005939.1 | Thao et al. (2004a) |
| *Aleurodicus rugioperculatus* | NC_082097.1 | Kumar et al. (2022) |
| *Bombyx mori C-108* | AB070264.1 | Yukuhiro et al. (2002) |
| *Rondotia menciana* | KT258908.1 | Kong et al. (2015) |
| *Pseudodendrothrips mori* | MN167468.1 | Unpublished data |

**References**

Kong W, Yang J. 2015. The complete mitochondrial genome of *Rondotia menciana* (Lepidoptera: Bombycidae). J Insect Sci. 15(1):48-56. doi: 10.1093/jisesa/iev032.

Kumar V, Pakrashi A, Kalleshwaraswamy CM, Banerjee D, Tyagi K. 2022. Gene rearrangement in the mitogenome of whiteflies (Hemiptera: Aleyrodinae) along with their phylogeny and characterization of complete mitogenome of *Aleurodicus rugioperculatus*. Mol Biol Rep. 49(6):4399-4409. doi: 10.1007/s11033-022-07275-7.

Lei T, Zhong YW, Liu YQ. 2021. Complete mitochondrial genome of the whitefly *Aleyrodes shizuokensis* Kuwana (Hemiptera: Aleyrodidae), new record from Chinese mainland. Mitochondrial B Resour. 6(2):397-398. doi: 10.1080/23802359.2020.1869617.

Lu MX, Chen ZT, Yu WW, Zhou DY. 2017. The complete mitochondrial genome of a spiraling whitefly, *Aleurodicus dispersus* Russell (Hemiptera: Aleyrodidae). Mitochondrial DNA A DNA Mapp Seq Anal. 28(2):165-166. doi: 10.3109/19401736.2015.1115492.

Thao M LL, Baumann L, Baumann P. 2004b. Organization of the mitochondrial genomes of whiteflies, aphids, and psyllids (Hemiptera, Sternorrhyncha). BMC Evol Biol. 4(1):1-13. doi: 10.1186/1471-2148-4-25.

Thao M LL, Baumann P. 2004a. Evolutionary relationships of primary prokaryotic endosymbionts of whiteflies and their hosts. Appl Environ Microbiol. 70(6):3401-3406. doi: 10.1128/AEM.70.6.3401-3406.2004.

Wang HL, Zhang Z, Bing XL, Liu YQ, Liu SS, Wang XW. 2016. A complete mitochondrial DNA genome derived from a Chinese population of the *Bemisia afer* species complex (Hemiptera: Aleyrodidae). Mitochondrial DNA A DNA Mapp Seq Anal. 27(5):3500-3501. doi: 10.3109/19401736.2015.1066367.

Yukuhiro K, Sezutsu H, Itoh M, Shimizu K, Banno Y. 2002. Significant levels of sequence divergence and gene rearrangements have occurred between the mitochondrial genomes of the wild mulberry silkmoth, *Bombyx mandarina*, and its close relative, the domesticated silkmoth, *Bombyx mori*. Mol Biol Evol. 19(8):1385-1389. doi: 10.1093/oxfordjournals.molbev.a004200.

Zhang ZT, Yan X, Yang WJ, Jin DC. 2020. Characterization of the complete mitochondrial genome of *Pealius machili* (Hemiptera: Aleyrodidae) with phylogenetic analysis. Mitochondrial DNA B Resour. 5(2):1463-1464. doi: 10.1080/23802359.2020.1741468.
