# Supplementary material for: The complete mitochondrial genome and phylogenetic analysis of Pealius mori (Hemiptera: Aleyrodidae)
Source: Mitochondrial DNA B Resour. 2024 Jul 5;9(7):856–60. doi: 10.1080/23802359.2024.2373229 (PMC11229734; doi:10.1080/23802359.2024.2373229)
Supplement: Supplemental Material [file TMDN_A_2373229_SM9475.doc]

**Table S1.** Comparison of nucleotide sequence homology (%) of 13 PCGs between two *P. mori* isolates

|  | ND1 | COX3 | ND3 | CYTB | ND6 | ND4L | ND4 | ND5 | ATP6 | ATP8 | COX2 | COX1 | ND2 |
| --- | --- | --- | --- | --- | --- | --- | --- | --- | --- | --- | --- | --- | --- |
| China | 100 | 100 | 99.7 | 100 | 99.8 | 100 | 100 | 100 | 99.7 | 100 | 100 | 89.3 | 100 |
| France |
