# Supplementary figures and images for: The complete mitochondrial genome and phylogenetic analysis of Pealius mori (Hemiptera: Aleyrodidae)
Source: Mitochondrial DNA B Resour. 2024 Jul 5;9(7):856–60. doi: 10.1080/23802359.2024.2373229 (PMC11229734; doi:10.1080/23802359.2024.2373229)

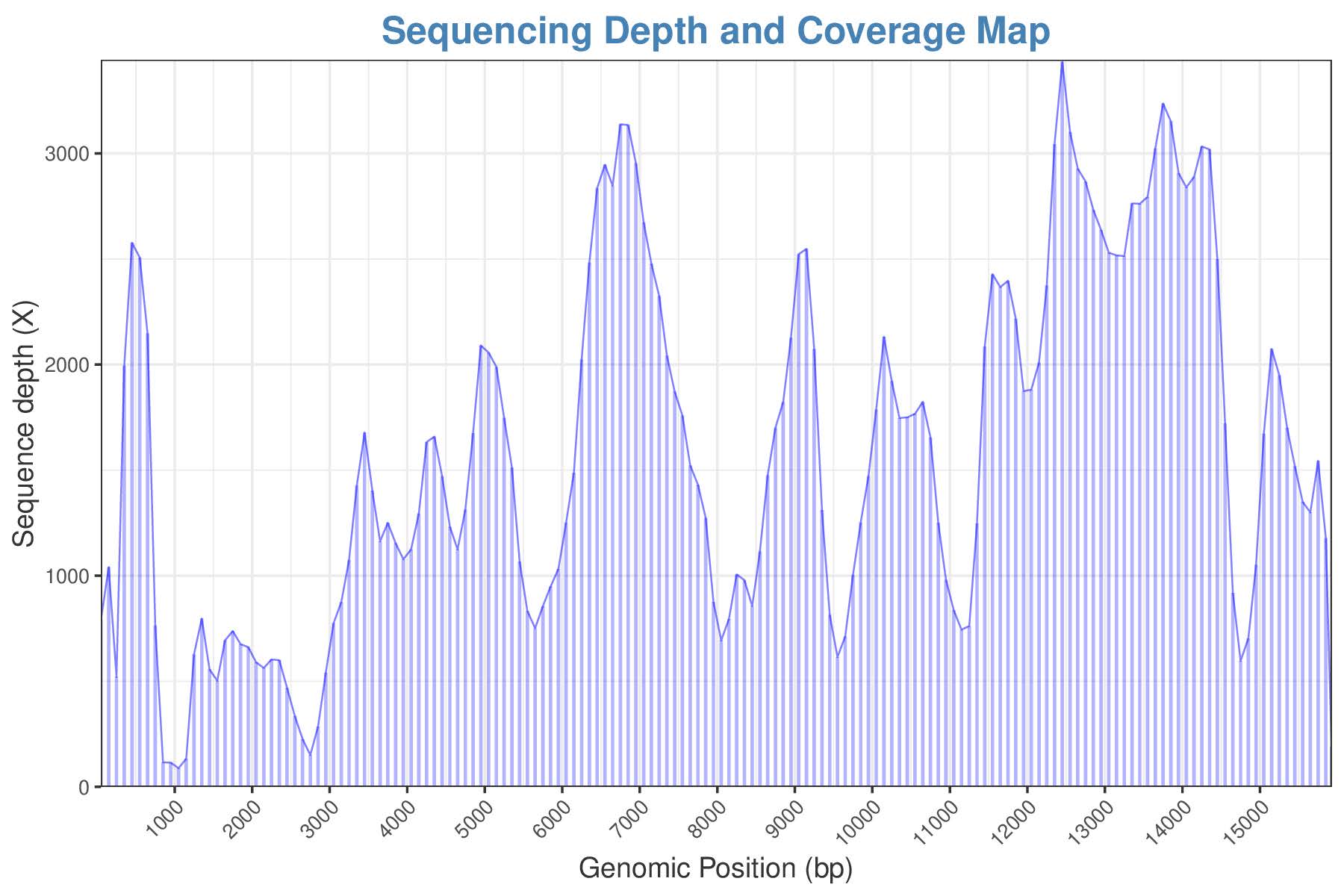

Supplement: Supplemental Material [file TMDN_A_2373229_SM9449.jpg]

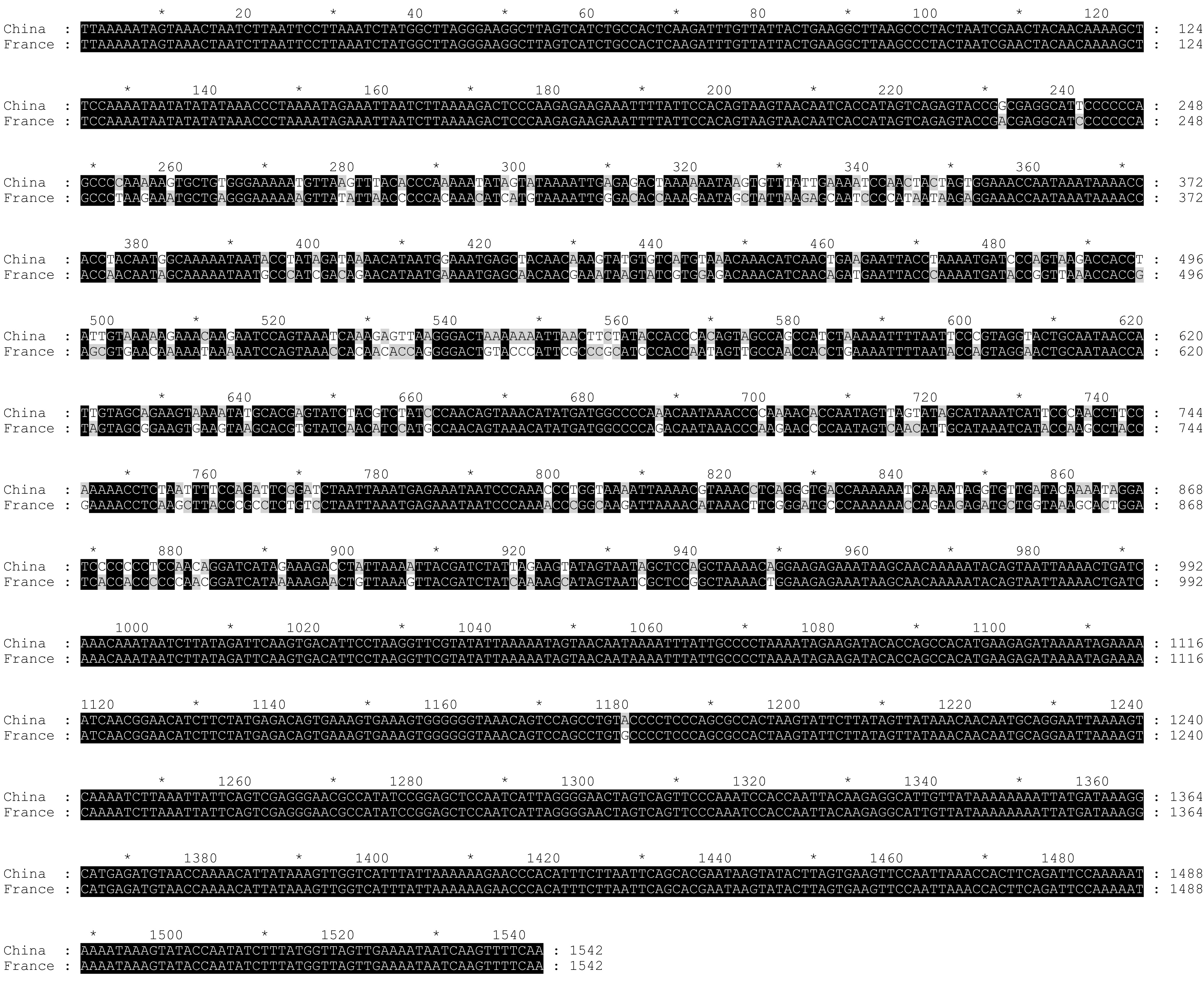

Supplement: Supplemental Material [file TMDN_A_2373229_SM9440.jpg]
